# Awaking Blood Pressure Surge and Progression to Microalbuminuria in Type 2 Normotensive Diabetic Patients

**DOI:** 10.1155/2016/5876792

**Published:** 2015-12-28

**Authors:** Michelangela Barbieri, Maria Rosaria Rizzo, Ilaria Fava, Celestino Sardu, Nicola Angelico, Pasquale Paolisso, Angela Abbatecola, Giuseppe Paolisso, Raffaele Marfella

**Affiliations:** ^1^Department of Medical, Surgical, Neurological, Metabolic and Geriatric Sciences, Second University of Naples, Piazza Miraglia 2, 80138 Naples, Italy; ^2^Evangelical Hospital Villa Betania, Naples, Italy; ^3^Alzheimer's Disease Clinic, ASL Frosinone, Atina, Italy

## Abstract

*Background*. We investigated the predictive value of morning blood pressure surge (MBPS) on the development of microalbuminuria in normotensive adults with a recent diagnosis of type 2 diabetes.* Methods*. Prospective assessments of 24-hour ambulatory blood pressure monitoring and urinary albumin excretion were performed in 377 adult patients. Multivariate-adjusted Cox regression models were used to assess hazard ratios (HRs) between baseline and changes over follow-up in MBPS and the risk of microalbuminuria. The MBPS was calculated as follows: mean systolic BP during the 2 hours after awakening minus mean systolic BP during the 1 hour that included the lowest sleep BP.* Results*. After a mean follow-up of 6.5 years, microalbuminuria developed in 102 patients. An increase in MBPB during follow-up was associated with an increased risk of microalbuminuria. Compared to individuals in the lowest tertile (−0.67 ± 1.10 mmHg), the HR and 95% CI for microalbuminuria in those in the highest tertile of change (24.86 ± 6.92 mmHg) during follow-up were 17.41 (95% CI 6.26–48.42); *p* for trend <0.001. Mean SD MBPS significantly increased in those who developed microalbuminuria from a mean [SD] of 10.6 [1.4] to 36.8 [7.1], *p* < 0.001.* Conclusion*. An increase in MBPS is associated with the risk of microalbuminuria in normotensive adult patients with type 2 diabetes.

## 1. Introduction

The development of microalbuminuria in type 2 diabetes defined as a urinary albumin excretion rate (UAER) of ≥20 *μ*g/min strongly predicts end stage renal disease and is also an independent risk factor for the development of cardiovascular disease, as well as cardiovascular mortality [1, 2]. In patients with type 2 diabetes and severe hypertension, the presence of microalbuminuria is a common finding [3]. Moreover, when hypertension is first detected in adults with type 2 diabetes, microalbuminuria is usually absent [4, 5]. Thus, one may hypothesize that microalbuminuria develops when hypertension is established. Interestingly, it has also been shown that the incidence of microalbuminuria among type 2 diabetic patients without hypertension was approximately 40% [6]. In a previous report, we found that increased MBPS and microalbuminuria in normotensive patients with type 2 diabetes were associated with an increased cardiovascular risk [7]. A recent study showed that normotensive subjects with type 2 diabetes and microalbuminuria had higher morning blood pressure than either of the normotensive subjects with type 2 diabetes and normal urinary albumin excretion or age-matched controls [8]. Such studies showed that persons with type 2 diabetes and microalbuminuria often have associated with higher morning blood pressure; however no studies have aimed at identifying if MBPS can predict the development of microalbuminuria in normotensive adults with diabetes. If an elevation in blood pressure, manifested as morning hypertension, precedes the development of microalbuminuria, this finding will provide an important starting point for treating susceptible patients before the onset of microalbuminuria. It is important to determine whether morning hypertension develops before microalbuminuria or, at the same time, in view of increasing evidence that the risk of progression to overt nephropathy is strongly influenced by the level of blood pressure, such as the degree of glycemic control [9]. Thus, we performed 24-hour ambulatory blood pressure monitoring in a prospective study of normotensive adults with type 2 diabetes and normal urinary albumin excretion at the time of enrollment.

## 2. Methods

### 2.1. Subjects and Study Design

We studied 377 newly diagnosed adults (age range 35–59 years) with type 2 diabetes from the outpatient clinics of Internal Medicine at the Second University in Naples, Italy, and from the Evangelical Hospital in Naples, Italy, between January 2001 to January 2010. All the subjects had been recently diagnosed with type 2 diabetes (less than 5 months) according to standard ADA criteria [10]. Inclusion criteria included normal urinary albumin excretion and arterial blood pressure (no previous use of antihypertensive agents). Normal urinary excretion from two separate 24-hour urine specimens was defined as 0 to 29 mg/24 hours. Normal blood pressure, measured at an office visit, was defined as ≤130/80 mmHg. Exclusion criteria included the presence of any clinical evidence of diabetic complications such as proliferative retinopathy, clinical and autonomic neuropathy, nephropathy, and/or ischemic cardiovascular disease. Subjects were followed prospectively, with measurement of urinary albumin excretion every six months. Those in whom microalbuminuria developed were withdrawn from the study, since they were generally given an angiotensin-converting-enzyme inhibitor as part of standard treatment at that point. At baseline, all patients completed a standardized questionnaire to assess patient clinical characteristics. Body mass index (BMI) was calculated as weight (kg) divided by height (m) squared. Waist circumference was measured at the midpoint between the lower rib margin and the iliac crest. Centralized laboratory biochemical analyses were performed on blood samples obtained after at least 12 hours of fasting. Plasma glucose was determined by an enzymatic colorimetric assay using a modified glucose-oxidase method. Glycated hemoglobin (A1c) was measured by high performance liquid chromatography using DCCT-aligned methods and standardized assays. Commercial enzymatic tests were used for determining serum total and high-density lipoprotein cholesterol and triglyceride levels. Urinary albumin excretion was measured in two separate 24-hour urine specimens with the use of a nephelometric immunoassay (Behring Institute; normal range, 0 to 29 mg/24 hours). Microalbuminuria was defined as a value for urinary albumin excretion that ranged from 30 to 299 mg/24 hours, as confirmed by two consecutive measurements less than six months apart. The ethical committee of Second University of Naples approved the study. All participants gave written informed consent.

### 2.2. Blood Pressure Measurements

A physician measured clinic BP with a mercury sphygmomanometer in the hospital clinic before the beginning of ABPM, with the subject sitting for at least 10 minutes. The average of 3 measurements was considered for analysis. The arm with higher BP values at office evaluations was chosen for the ABPM, which was performed with DynaPulse 5000A (Pulse Metric, Inc.). To reduce errors during the measurements caused by the position of the upper arm during the day, we asked all subjects to ensure that the arm was always parallel to the trunk when the cuff was inflated. Recording began between 8:30 and 9 a.m. The pressure was measured at 30-minute intervals for 24 hours. We excluded patients who obtained less than 80% of either awake or asleep valid BP readings (*n* = 10). Patients who reported in our post-ABPM questionnaire that their sleep was severely disturbed by wearing the ABPM were also excluded from this study (*n* = 12). The morning blood pressure surge (MBPS) was calculated as follows: mean systolic BP during the 2 hours after awakening minus mean systolic BP during the 1 hour that included the lowest sleep BP [11, 12]. The systolic pressures were used for all these calculations. Subjects with a nocturnal reduction of systolic and/or diastolic BP ≥ 10% were defined as dippers and the others as nondippers. Nighttime workers, subjects going to bed later than 1:00 a.m., and patient with sleep disordered breathing were excluded from the present study; therefore, all study subjects were in bed during the entire nighttime period and were awake and active during the morning and daytime interval.

### 2.3. Statistical Analysis

Statistical analyses were performed using SPSS software (Chicago, IL). All data are presented as mean ± standard deviation (SD), unless otherwise indicated. Analysis of variance (ANOVA) test for continuous variables and the *χ* ^2^ test for categorical variables were used to evaluate differences in clinical characteristics across baseline MBPS tertiles. Survival analysis through the observation period was performed using the Kaplan-Meier. Multivariate Cox proportional regression models were used to assess the relative risk of microalbuminuria according to MBPS tertiles at baseline. Additional Cox regression models were used to assess the association between the changes in MBPS during the follow-up and incidence of microalbuminuria. Changes in MBPS were calculated from baseline to the end of the follow-up (microalbuminuria diagnosis). Regression models were adjusted for potential confounders. The first model was adjusted for age, gender, BMI, and also baseline MBPS when analyzing the association between the changes in MBPS levels during follow-up. The second model included the same confounders in Model 1 and glycated hemoglobin, duration of diabetes, total cholesterol, triglycerides, and fasting plasma glucose. The time variable was the interval between enrolment and the date of microalbuminuria diagnosis or the date of the last visit for patients who remained normoalbuminuric.

## 3. Results

At the time of enrolment, urinary albumin excretion was normal (13.3 ± 3.4 mg/24 hours). Mean systolic and diastolic BPs were 122 ± 8 mmHg and 79 ± 7 mmHg, respectively. The mean values for 24-hour ambulatory blood pressure were in the normal range (daytime systolic and diastolic pressure, 120 ± 8 and 75 ± 6 mmHg, resp.; nighttime systolic and diastolic pressure, 100 ± 5 and 59 ± 4 mmHg, resp.); MBPS was 10.8 ± 1.5 mmHg. All patients were dippers.

The baseline characteristics of the total study population (*n* = 377) according to tertiles of MBPS (mmHg) are reported in Table 1. There were not any significant differences according to fasting or postprandial glucose, HDL, systolic or diastolic blood pressures, or 24-hour urinary albumin excretion. Mean values in the lowest and highest MBPS tertile were 9.5 mmHg and 12.5, respectively. We found that there was a greater number of women in the highest tertile of MBPS (*p* = 0.012).

### 3.1. Baseline Characteristics according to the Development of Microalbuminuria

After a mean follow-up of 78.8 ± 5.2 months, microalbuminuria developed in 102 of the 377 subjects (27%); 24-hour urinary albumin excretion in this group increased from 13.8 ± 3.4 to 109.1 ± 38.4 mg (*p* < 0.001) (Table 2). Normoalbuminuric patients (*n* = 275) also had a significant increase in 24-hour urinary albumin excretion that remained in a normal range of urinary albumin excretion (from 13.3 ± 3.4 to 19.9 ± 22.6 mg, *p* < 0.001). Baseline characteristics according to the development of microalbuminuria are reported in Table 2. Both groups were similar with respect to age, sex distribution, BMI, HbA1c, mean systolic and diastolic blood pressure, MBPS, and urinary albumin excretion (Table 2). Even though baseline systolic blood pressure was slightly higher in the microalbuminuric (MA) compared to the normoalbuminuric (NA) group, it was not statistically significant (*p* = 0.053). The nocturnal reduction of systolic and/or diastolic BP was >10% in all patients. In addition, there were not significant differences at baseline in heart rate, ambulatory systolic and diastolic pressure measures, and daytime and nighttime systolic and diastolic blood pressures between groups (data not shown).  Table 3 presents the hazard ratios (HRs) and 95% CIs for microalbuminuria according to baseline MBPS. We did not find any significant risk for developing microalbuminuria in models adjusted for age, gender, and BMI (HR 1.56 [95% CI 0.58–4.2]); *p* for trend = 0.26. Even though the fully adjusted model showed an increasing trend of HR across tertiles of MBPS, this trend was not significant (HR 1.89 [95% CI 0.68–5.23]).

### 3.2. Changes in MBPS during Follow-Up and Risk of Microalbuminuria

**Figure 1:**
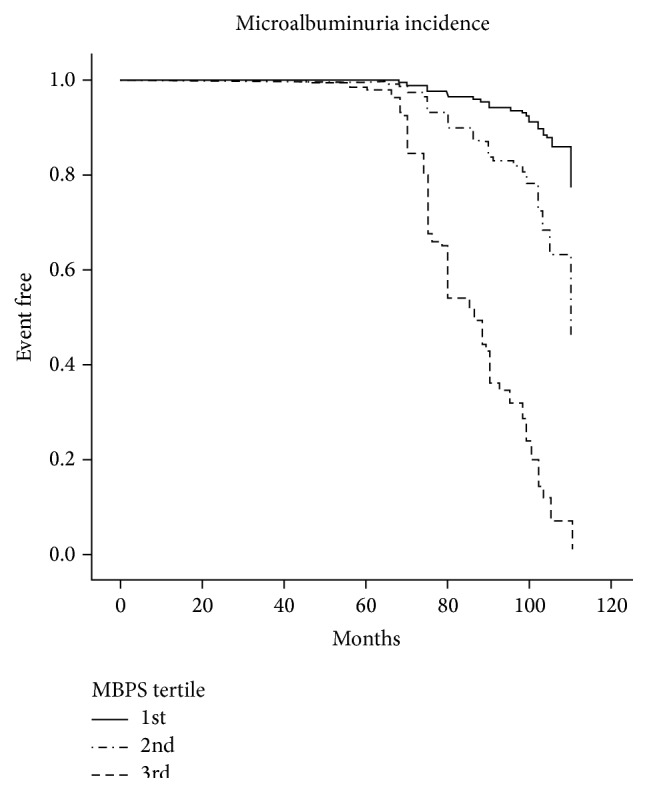
Kaplan-Meier curves were used to illustrate time to event of microalbuminuria according to the tertile change of MBPS.

102 patients that developed microalbuminuria had a significant increase in MBPS over time (from 10.9 ± 1.4 to 35.3 ± 9.2 mmHg), while the MBSP did not significantly change in those who remained normoalbuminuric over time (Table 2) (Figure 1).

Table 4 shows the HRs and 95% CIs for microalbuminuria according to the changes in MBPS during follow-up. After adjusting for multiple confounders, in the fully adjusted model 2, the risk of microalbuminuria in patients with the highest tertile of change (24.86 mmHg) was at least 6 times more likely compared to those in the lowest tertile change. This finding suggests that an increase in MBPS of at least 25 mmHg is at least 6 times more likely to be associated with the development of microalbuminuria in adults with type 2 diabetes.

Lastly, proliferative retinopathy developed in 18 subjects (5 of 106 in the microalbuminuria group and 13 of 271 in the normoalbuminuria group), but none of the 18 subjects had clinical evidence of neuropathy.

## 4. Discussion

Hypertension is certainly a major determinant of microangiopathy in diabetes, but the relationship between hypertension and microalbuminuria in diabetes is complex. Hypertension and microalbuminuria often coexist in diabetic patients, and reducing blood pressure reduces microalbuminuria in type 1 diabetes [13]. However, it is unclear whether hypertension contributes to the development of microalbuminuria in type 2 diabetes. At least in type 1 diabetes, alteration of circadian blood pressure appears to precede the microalbuminuria: in longitudinal studies there is evidence that an increase in systolic blood pressure during sleep precedes the development of microalbuminuria [14]. This is more difficult to demonstrate in type 2 diabetes, perhaps because of the heterogeneity of the disease. In this study, which involved a cohort of newly diagnosed patients with type 2 diabetes, an increase in blood pressure during morning period, detected by ambulatory blood pressure monitoring, preceded the development of microalbuminuria. In contrast, in subjects in whom microalbuminuria did not develop during a follow-up period of more than five years, neither nighttime, daytime, nor morning blood pressure increased significantly. Thus, the risk of microalbuminuria, a marker of kidney disease in patients with type 2 diabetes, appears to be very low in patients who remain normotensive, as defined not only by normal blood pressure readings at office visits and during ambulatory daytime monitoring over time but also by the absence of an increase in systolic pressure during the morning period. Moreover, we evidenced that neither office visit blood pressure evaluations nor daytime blood pressure, during ambulatory blood pressure monitoring, changed significantly over time (i.e., during the period of normoalbuminuria) in subjects in whom microalbuminuria developed. Thus, morning-time systolic pressure increase seems to be the first demonstrable manifestation of altered blood pressure regulation in patients with type 2 diabetes. Our study evidenced a chronological relation between an increase in morning blood pressure and the development of microalbuminuria in patients with type 2 diabetes. Thus, an early increase in morning-time arterial blood pressure may have a central role in the development of diabetic nephropathy. In this context, systemic pressure overload, initially limited to systolic pressure during morning period, when transferred to the glomerular circulation, may cause hemodynamic changes in the kidney, conducting microalbuminuria, structural kidney damage, or both. Our results fit with the hypothesis that a predisposition to essential hypertension increases the risk of diabetic nephropathy. This hypothesis is supported by studies indicating that the parents of patients with type 2 diabetes and proteinuria have a higher prevalence of hypertension than that in the general population [15, 16]. On the other hand, many studies evidenced that microalbuminuria is strongly influenced by the level of glycosylated hemoglobin [17]. However, previous studies evidenced that microalbuminuria did not develop in patients with very poor metabolic control in whom blood pressure remained normal [17]. Although poor metabolic control accelerates the progression of kidney disease [18], in our study, as evidenced by Cox proportional hazard ratio for microalbuminuria incidence according to changes in MBPS, nephropathy appears to develop mainly in susceptible persons with type 2 diabetes and altered blood pressure circadian rhythm. Since slight but timely increase in blood pressure comes before the development of microalbuminuria, we suppose that, despite of the level of metabolic control, an increase in morning blood pressure plays a key role in the development of microalbuminuria in type 2 diabetes. Several possible mechanisms underlying the relationship between morning blood pressure increase and elevated albuminuria are postulated in the previous studies. Knudsen et al. [19] demonstrated the presence of strong associations between elevated plasma levels of proteins, including E-selectin and intercellular adhesion molecule 1, related to endothelial activation by an augmentation of MBPS in patients with type 2 diabetes. The other report also indicated that MBPS is inversely correlated with endothelium-dependent acetylcholine-stimulated vasodilation in hypertensive patients [20]. Amar et al. [21] suggested elevation in MBPS was associated with systemic inflammation as measured by serum levels of C-reactive protein. These findings indicate that endothelial dysfunction and inflammation could represent a pathophysiological link between an elevated MBPS and microvascular complications. Thus, an evaluation of the blood pressure circadian rhythm at an early stage of type 2 diabetes would provide the best basis for choosing therapies designed to prevent the progression to microalbuminuria. The increase in morning blood pressure has been associated with cardiovascular complications in subjects with essential hypertension [22] and, more recently, in those with type 2 diabetes and overt nephropathy [8].

## 5. Conclusion

Early documentation of an increase in morning pressure might warrant the use of agents such as angiotensin-converting enzyme inhibitors or angiotensin II-receptor blockers in a patient with type 2 diabetes. Documentation of normal morning blood pressure, on the other hand, might suggest that there is no need for early therapeutic interventions other than those designed to provide optimal glycemic control.

## Novelty and Significance

*What Is New?* We for the first time evidenced that an increase in blood pressure during morning period, detected by ambulatory blood pressure monitoring, preceded the development of microalbuminuria in newly diagnosed type 2 diabetes. In contrast, in subjects in whom microalbuminuria did not develop during a follow-up period of more than five years, neither nighttime, daytime, nor morning blood pressure increased significantly.

*What Is Relevant?* The evaluation of the blood pressure circadian rhythm at an early stage of type 2 diabetes would provide the best basis for choosing therapies designed to prevent the progression to microalbuminuria. Early documentation of an increase in morning pressure might warrant the use of agents such as angiotensin-converting enzyme inhibitors or angiotensin II-receptor blockers in a patient with type 2 diabetes. 

*Summary*. An increase in morning systolic blood pressure is associated with the risk of microalbuminuria in normotensive adult patients with type 2 diabetes.

## Figures and Tables

**Table 1:** Characteristics of the study population according to mean morning blood pressure surge (MBPS) values at baseline (*n* = 377).

	All	Tertiles of MBPS (mmHg)			*p*
		1	2	3	
*n*	377	167	82	128	
MBPS (mmHg)	10.8 ± 1.5	9.5 ± 0.7	11.0 ± 0.0	12.5 ± 0.7	
Age (years)	50 ± 6	50 ± 6	51 ± 5	50 ± 6	0.906
Gender (m/f)	194/183	84/56	45/40	65/87	0.012
BMI (kg/m^2^)	29.8 ± 2.0	30.0 ± 2.0	29.6 ± 2.0	29.8 ± 2.0	0.342
Fasting plasma glucose (mg/dL)	206 ± 41	205 ± 46	210 ± 37	206 ± 37	0.573
Postprandial glucose (mg/dL)	211 ± 35	210 ± 36	213 ± 34	212 ± 33	0.734
HbA1c (%)	9.4 ± 1.3	9.4 ± 1.3	9.7 ± 1.3	9.4 ± 1.4	0.199
HDL-cholesterol (mg/dL)	38.0 ± 3.6	37.6 ± 3.4	38.1 ± 3.7	38.4 ± 3.6	0.114
Daytime systolic BP (mmHg)	122 ± 8	123 ± 9	121 ± 8	122 ± 8	0.622
Nighttime systolic BP (mmHg)	100 ± 5	99 ± 5	100 ± 5	100 ± 5	0.334
Daytime diastolic BP (mmHg)	79 ± 7	79 ± 7	79 ± 6	78 ± 7	0.901
Nighttime diastolic BP (mmHg)	59 ± 4	59 ± 4	59 ± 43	58 ± 4	0.463
Urinary albumin excretion (mg/24 hours)	13.4 ± 3.4	13.5 ± 3.5	13.9 ± 3.6	12.9 ± 3.2	0.152

Data are presented as means ± SD unless otherwise indicated. BMI: body mass index; BP: blood pressure.

**Table 2:** Clinical characteristics according to the development of microalbuminuria at baseline and the last follow-up (*n* = 377).

		Baseline			Follow-up	
	NA	MA	*p*	NA	MA	*p*
*n*	102	275		102	275	
Age (years)	50 ± 6	51 ± 6	0.466	57 ± 6^*∗*^	55 ± 7^§^	0.008
Gender (m/f)	144/131	50/52	0.565	144/131	50/52	0.565
BMI (kg/m^2^)	29.8 ± 2.0	30.0 ± 2.2	0.322	29.9 ± 2.0	29.4 ±2.0	0.016
HbA1c (%)	9.4 ± 1.3	9.5 ± 1.4	0.469	7.2 ± 1.0^*∗*^	7.4 ± 0.8^§^	0.134
Heart rate	77 ± 9	76 ± 9	0.388	79 ± 10	78 ± 11	0.597
Mean systolic BP (mmHg)	122 ± 8	123 ± 10	0.053	126 ± 10^*∗*^	125 ± 11	0.707
Mean diastolic BP (mmHg)	79 ± 7	78 ± 6	0.230	79 ± 11	79 ± 8	0.951
MBSP (mmHg)	10.8 ± 1.5	10.9 ± 1.4	0.721	11.7 ± 2.2	35.3 ± 9.2^^§^^	<0.001
Urinary albumin excretion (mg/24 hours)	13.3 ± 3.4	13.8 ± 3.4	0.204	19.9 ± 22.6^*∗*^	109 ± 38.4^§^	<0.001

*p*: comparison between NA and MA performed separately at baseline and at the last follow-up. *∗*: *p* < 0.05 NA at baseline versus follow-up; §: *p* < 0.05 MA at baseline versus MA at follow-up; MA: microalbuminuric; NA: normoalbuminuric.

**Table 3:** Cox proportional hazard ratio (95% CI) for microalbuminuria incidence according to baseline MBPS.

	Tertiles of morning blood pressure surges, mmHg			*p* for trend
	1 (low)	2	3	
*n*	167	82	128	
Mean MBPS	9.5 (0.7)	11.0 (0.0)	12.5 (0.7)	
Microalbuminuria, *n* (%)	15 (14.7)	27 (26.5)	60 (58.8)	
Model 1	1 (ref.)	1.90 (0.92–3.93)	1.74 (0.65–4.66)	0.22
Model 2	1 (ref.)	1.91 (0.89–4.07)	2.054 (0.74–5.72)	0.25

Data are presented as mean ± SD unless otherwise indicated. Cox regression models were used to assess the risk of developing microalbuminuria by mean morning blood pressures surges (MBPS) at baseline. Model 1: adjusted for age, gender, BMI, and *SBP*. Model 2: Model 1 + glycated hemoglobin, duration of diabetes, total cholesterol, triglycerides, and fasting plasma glucose.

**Table 4:** Cox proportional hazard ratio (95% CI) for microalbuminuria incidence according to changes in MBPS.

	Tertiles of changes in morning blood pressure surges, mmHg			*p* for trend
	1 (low)	2	3	
*n*	176	72	129	
Mean change MBPS	−0.67 (1.10)	2.41 (1.21)	24.86 (6.92)	
Microalbuminuria, *n* (%)	4 (2.3)	9 (12.5)	89 (69)	
Model 1	1 (ref.)	**3.87 (1.18–12.72)**	**14.48 (5.25–39.92)**	**0.026**
Model 2	1 (ref.)	**4.83 (1.46–16.03)**	**16.76 (6.07–46.32)**	**<0.001**

Data are presented as mean ± SD unless otherwise indicated. Cox regression models were used to assess the risk of developing microalbuminuria by changes in morning blood pressures surges (MBPS). Model 1: adjusted for age, gender, BMI, baseline MBPS, and *baseline SBP*. Model 2: Model 1 + glycated hemoglobin, duration of diabetes, total cholesterol, triglycerides, and fasting plasma glucose.

## References

[1] Sowers J. R., Epstein M., Frohlich E. D. (2001). Diabetes, hypertension, and cardiovascular disease: an update. *Hypertension*.

[2] Bilous R., Chaturvedi N., Sjølie A. K. (2009). Effect of candesartan on microalbuminuria and albumin excretion rate in diabetes: three randomized trials. *Annals of Internal Medicine*.

[3] Parving H.-H., Lehnert H., Bröchner-Mortensen J., Gomis R., Andersen S., Arner P. (2001). The effect of irbesartan on the development of diabetic nephropathy in patients with type 2 diabetes. *The New England Journal of Medicine*.

[4] Ritz E., Orth S. R. (1999). Nephropathy in patients with type 2 diabetes mellitus. *The New England Journal of Medicine*.

[5] Parving H.-H., Lehnert H., Brochner-Mortensen J., Gomis R., Andersen S., Arner P. (2001). The effect of irbesartan on the development of diabetic nephropathy in patients with type 2 diabetes. *The New England Journal of Medicine*.

[6] Basi S., Fesler P., Mimran A., Lewis J. B. (2008). Microalbuminuria in type 2 diabetes and hypertension: a marker, treatment target, or innocent bystander?. *Diabetes Care*.

[7] Marfella R., Esposito K., Giugliano D. (2003). Increase in nocturnal blood pressure and progression to microalbuminuria in diabetes. *The New England Journal of Medicine*.

[8] Ushigome E., Fukui M., Hamaguchi M. (2013). Morning pulse pressure is associated more strongly with elevated albuminuria than systolic blood pressure in patients with type 2 diabetes mellitus: post hoc analysis of a cross-sectional multicenter study. *Diabetes Research and Clinical Practice*.

[9] Zoppini G., Targher G., Chonchol M. (2012). Predictors of estimated GFR decline in patients with type 2 diabetes and preserved kidney function. *Clinical Journal of the American Society of Nephrology*.

[10] American Diabetes Association (2002). Standards of medical care for patients with diabetes mellitus. *Diabetes Care*.

[11] Kario K., Pickering T. G., Umeda Y. (2003). Morning surge in blood pressure as a predictor of silent and clinical cerebrovascular disease in elderly hypertensives: a prospective study. *Circulation*.

[12] Marfella R., Gualdiero P., Siniscalchi M. (2003). Morning blood pressure peak, QT intervals, and sympathetic activity in hypertensive patients. *Hypertension*.

[13] The ACE Inhibitors in Diabetic Nephropathy Trialist Group (2001). Should all patients with type 1 diabetes mellitus and microalbuminuria receive angiotensin-converting enzyme inhibitors?. *Annals of Internal Medicine*.

[14] Lurbe E., Redon J., Kesani A. (2002). Increase in nocturnal blood pressure and progression to microalbuminuria in type 1 diabetes. *The New England Journal of Medicine*.

[15] Crispim D., Canani L. H., Gross J. L., Tschiedel B., Souto K. E. P., Roisenberg I. (2006). Familial history of type 2 diabetes in patients from southern Brazil and its influence on the clinical characteristics of this disease. *Arquivos Brasileiros de Endocrinologia e Metabologia*.

[16] Charles M. A., Pettitt D. J., Hanson R. L. (1994). Familial and metabolic factors related to blood pressure in Pima Indian children. *American Journal of Epidemiology*.

[17] Coca S. G., Ismail-Beigi F., Haq N., Krumholz H. M., Parikh C. R. (2012). Role of intensive glucose control in development of renal end points in type 2 diabetes mellitus: systematic review and meta-analysis intensive glucose control in type 2 diabetes. *Archives of Internal Medicine*.

[18] Adler A. I., Stevens R. J., Manley S. E., Bilous R. W., Cull C. A., Holman R. R. (2003). Development and progression of nephropathy in type 2 diabetes: the United Kingdom Prospective Diabetes Study (UKPDS 64). *Kidney International*.

[19] Knudsen S. T., Jeppesen P., Frederiksen C. A. (2007). Endothelial dysfunction, ambulatory pulse pressure and albuminuria are associated in type 2 diabetic subjects. *Diabetic Medicine*.

[20] Ceravolo R., Maio R., Pujia A. (2003). Pulse pressure and endothelial dysfunction in never-treated hypertensive patients. *Journal of the American College of Cardiology*.

[21] Amar J., Ruidavets J.-B., Sollier C. B. D. (2004). Relationship between C reactive protein and pulse pressure is not mediated by atherosclerosis or aortic stiffness. *Journal of Hypertension*.

[22] Luo Y., Wang Y.-L., Wu Y.-B. (2013). Association between the rate of the morning surge in blood pressure and cardiovascular events and stroke. *Chinese Medical Journal*.

